# Biosynthetic gene clusters from uncultivated soil bacteria of the Atacama Desert

**DOI:** 10.1128/msphere.00192-24

**Published:** 2024-09-17

**Authors:** Constanza M. Andreani-Gerard, Verónica Cambiazo, Mauricio González

**Affiliations:** 1 Millennium Institute Center for Genome Regulation (CRG); 2 Bioinformatic and Gene Expression Laboratory, Instituto de Nutrición y Tecnología de los Alimentos (INTA), Santiago, Chile; 3 Center for Mathematical Modeling (CMM) – Universidad de Chile, Santiago, Chile; Kansas State University, Manhattan, Kansas, USA

**Keywords:** metagenomics, specialized metabolism, Atacama Desert

## Abstract

Soil microorganisms mediate several biological processes through the secretion of natural products synthesized in specialized metabolic pathways, yet functional characterization in ecological contexts remains challenging. Using culture-independent metagenomic analyses of microbial DNA derived directly from soil samples, we examined the potential of biosynthetic gene clusters (BGCs) from six bacterial communities distributed along an altitudinal gradient of the Andes Mountains in the Atacama Desert. We mined 38 metagenome-assembled genomes (MAGs) and identified 168 BGCs. Results indicated that most predicted BGCs were classified as non-ribosomal-peptides (NRP), post-translational modified peptides (RiPP), and terpenes, which were mainly identified in genomes of species from *Acidobacteriota* and *Proteobacteria* phyla. Based on BGC composition according to types of core biosynthetic genes, six clusters of MAGs were observed, three of them with predominance for a single phylum, of which two also showed specificity to a single sampling site. Comparative analyses of accessory genes in BGCs showed associations between membrane transporters and other protein domains involved in specialized metabolism with classes of biosynthetic cores, such as resistance-nodulation-cell division (RND) multidrug efflux pumps with RiPPs and the iron-dependent transporter TonB with terpenes. Our findings increase knowledge regarding the biosynthetic potential of uncultured bacteria inhabiting pristine locations from one of the oldest and driest nonpolar deserts on Earth.

**IMPORTANCE:**

Much of what we know about specialized metabolites in the Atacama Desert, including Andean ecosystems, comes from isolated microorganisms intended for drug development and natural product discovery. To complement research on the metabolic potential of microbes in extreme environments, comparative analyses on functional annotations of biosynthetic gene clusters (BGCs) from uncultivated bacterial genomes were carried out. Results indicated that in general, BGCs encode for structurally unique metabolites and that metagenome-assembled genomes did not show an obvious relationship between the composition of their core biosynthetic potential and taxonomy or geographic distribution. Nevertheless, some members of *Acidobacteriota* showed a phylogenetic relationship with specific metabolic traits and a few members of *Proteobacteria* and *Desulfobacterota* exhibited niche adaptations. Our results emphasize that studying specialized metabolism in environmental samples may significantly contribute to the elucidation of structures, activities, and ecological roles of microbial molecules.

## INTRODUCTION

Desert soils are complex environments, characterized by a very low ratio of mean annual precipitation (MAP) to potential evapotranspiration (PET; MAP/PET), a high level of solar radiation, and restricted vegetation covers (1). As arid environments display scarce vegetation, bacteria are essential for nutrient cycling and carbon storage (2, 3). In addition, microorganisms produce diverse specialized metabolites, also referred to as “secondary metabolites” or “natural products,” such as pigments, antibiotics, antifungals, and siderophores (4, 5). These compounds are involved in sensing the environment and in triggering several survival strategies, such as nutrient acquisition, motility and biofilm production, growth inhibition, and tolerance mechanisms against abiotic stresses (6).

Given that specialized metabolic pathways closely refer to niche adaptation, they show more restricted taxonomic distributions and admit a greater metabolic diversity than those involved in primary metabolism (7). Many pathways involved in specialized metabolism include enzymes with non-strict substrate specificity that produce a range of slightly different molecules (8). Such catalytic promiscuity enables the diversification of specialized metabolites and provides them more flexibility to respond to demanding and evolving environments (9). In fact, harsh environmental conditions, such as desert soils, have been suggested to select for microorganisms producing novel chemicals (10, 11). Moreover, specialized metabolites have been proposed as one of the factors that could modulate bacterial community assembly and spatial distribution in soils (12, 13). Hence, it is possible to associate these functional traits with the biogeochemical cycling of macronutrients and micronutrients that are required for the growth of plants and animal life in this extreme environment (14). For example, siderophores enhance iron uptake in natural environments, where the bioavailability of these metals is limited, increasing the nutrient turnover of soil microbial communities (15).

The biosynthesis of specialized metabolites is, almost always, governed by genes that are clustered in close physical proximity in the bacterial genome, known as biosynthetic gene clusters (BGCs) (16). The structural modularity of BGCs encoding for core and accessory enzymes reflects evolutionary history throughout horizontal transfer and seems to foster rapid evolution (17). Due to the diverse array of thriving mechanisms, classification of enzymes and comparative approaches are indispensable to assess specialized metabolism in evolutionary and ecological contexts (18, 19). Main classes of BGCs are assigned according to a few clearly defined types of core biosynthetic enzymes, making it data of predictive quality (20). Thereby, genome mining of BGCs has been established as a critical step in almost every current bioinformatics pipeline dedicated to specialized metabolites research and has proven to deliver significant insights into molecular mechanisms even when counting on *in silico* approaches only (21, 22). Recently, studies have started utilizing profiles of BGCs and other functional features to unveil ecological inferences through their correlation with biological and environmental data. For example, *via* multivariate analyses and an environment-wide association survey approach on community-weighted genomic traits, PFAM and COG entries, genome size, CRISPR arrays, and BGCs have been employed for defining “functional signatures” and bacterial “bioindicators” of health ratings in octocoral microbiomes and agricultural soils, respectively (23, 24).

In general, microorganisms isolated from extreme habitats, such as non-polar arid deserts, are considered a significant reservoir in the search and discovery of novel compounds (25, 26). In particular, studies of bioprospecting specialized metabolites in the Atacama Desert, one of the driest deserts on Earth, have been carried out using pure cultures (27–29). Rateb et al. (30) discuss the discovery and characterization of 46 specialized metabolites from actinobacterial strains isolated from various locations in this extreme environment (30). Considering that most desert microorganisms cannot be cultured, the reconstruction of metagenome-assembled genomes (MAGs) represents an alternative experimental approach to complement the research on metabolic potential (31). To date, metagenomic explorations in desert soils including the Atacama Desert have captured a significant amount of taxonomic and functional diversity of microbial populations (32–36). However, to our knowledge, analyses of the biosynthetic potential of specialized metabolites in uncultured soil bacteria from the Atacama Desert have not been carried out. This study aimed to determine the BGC composition of bacterial MAGs generated from six metagenomic samples spanning an altitudinal gradient that exhibited contrasting features like temperature, precipitation, soil nutrients, and vegetation belts of the Andes Mountains in the Atacama Desert.

## MATERIALS AND METHODS

### Sites and sample collection

**Fig 1:**
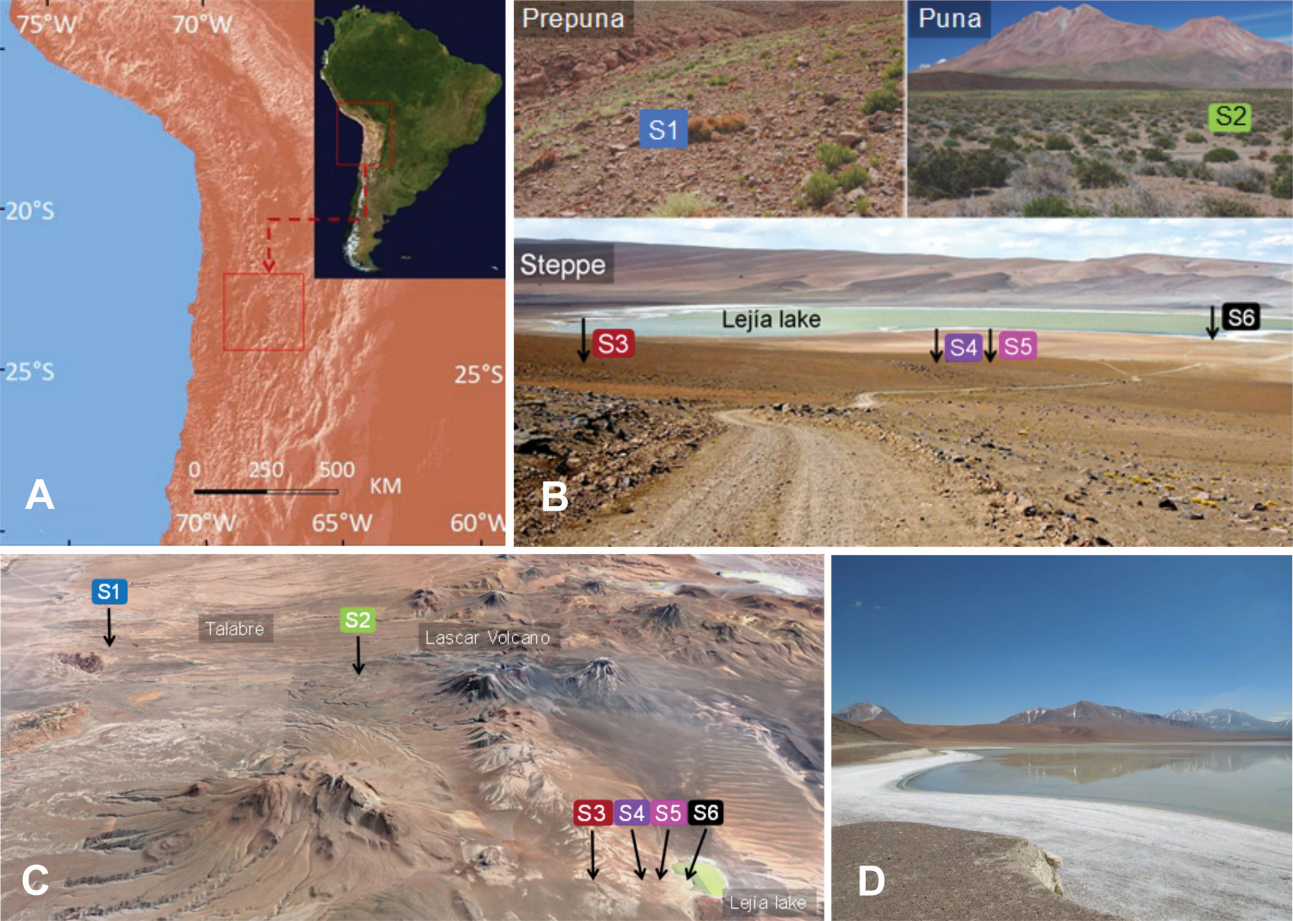
Environmental heterogeneity across the Talabre-Lejía transect (TLT). (**A**) Regional context of the studied sites in northern Chile. (**B**) Photograph of the landscape where sites S1 to S6 were sampled at the TLT. (**C**) Map of the transect created with ArcGis 10.8 using STRM3054,55 elevation model (Data: SIO, NOAA, U.S. Navy, NGA, GEBCO) and Landsat 8 Satellite image (data available from the U.S. Geological Survey). (**D**) Enlarged image of S6, sampled from the Lejía Lake’s shore.

Bulk soils were sampled during April 2014 after the rainy season at six sites along the Talabre-Lejía transect (TLT; ~23.4°S, ~67.8°W), located in the western Andes Mountain Range of Chile (Fig. 1). The altitudinal gradient covers the eastern margin of the Atacama Salar to the Lejía Lake [from 2,400 to 4,500 meters above sea level (m.a.s.l.)]. Sites were selected to span low, medium, and high elevations, and different characteristic vegetations: pre-puna (2,400 to 3,300 m.a.s.l.), puna (3,200 to 4,000 m.a.s.l.), and steppe (4,000 to 4,500 m.a.s.l.) (32) (Table S1). Soil samples (100 g) were collected in triplicate at a depth of 10 cm from the ground and stored in dry ice until arrival at the laboratory for metagenomic sequencings.

### Soil physicochemical and nutrient properties

The mean annual precipitation (MAP) and mean annual temperature (MAT) data were obtained from Díaz et al. (Table S1) (37). Soil pH was determined in a 1:1 wt/wt soil deionized water ratio and electrical conductivity in a 1:5 wt/wt soil deionized water ratio (38). A portion of the bulk soil samples was used to determine the metal composition using the total reflection X-ray fluorescence (TXRF) technique following a previously published protocol (39). Briefly, for each sample, 1 g of soil was resuspended in 1 mL of distilled water and homogenized for 2 h at room temperature. After mixing, the samples were centrifuged at 11,440 *g* for 10 min in a Hettich Universal 32R. The soluble fraction was recovered and measured in a Bruker S2 PICOFOX. Information on nutrient and physicochemical data were included in Table 1.

**TABLE 1:** Nutritional and physicochemical information of soil samples from S1 to S6. Nutrients are in mg/kg unless otherwise indicated. Each value is the average ± standard deviation of triplicate^*a*^

Nutrient data	S1	S2	S3	S4	S5	S6
pH	8.1 ± 0.3	6.1 ± 0.15	5.82 ± 0.09	7.54 ± 0.12	8.485 ± 0.01	8.52 ± 0.02
EC (mS/cm)	0.06 ± 0.01	0.1 ± 0.02	0.05 ± 0.01	0.06 ± 0	0.13 ± 0	1.94 ± 0.15
OM (%)	0.25 ± 0.08	1.02 ± 0.03	0.24 ± 0.03	0.17 ± 0.01	0.245 ± 0.01	0.485 ± 0.04
C	ND	ND	2,600 ± 100	2,400 ± 0	3,750 ± 250	10,650 ± 350
N	40 ± 10	60 ± 30	24 ± 0	25.5 ± 1.5	23 ± 4	29 ± 4
NH_4_	3.5 ± 1.5	9.6 ± 2.4	5 ± 0	13 ± 0	8.5 ± 1.5	8.5 ± 1.5
NO_3_	0.85 ± 0.15	5.5 ± 1.5	19 ± 0	12.5 ± 1.5	14.5 ± 2.5	20.5 ± 5.5
P	4 ± 1	21 ± 1	11.5 ± 2.5	6 ± 0	4.5 ± 0.5	59 ± 2
K	264 ± 105	148 ± 16	74 ± 8	146 ± 3	158.5 ± 3.5	770 ± 30
Mg	116 ± 7	57.1 ± 30.4	77.79 ± 14.49	344 ± 8.5	136.1 ± 9.7	1362 ± 81
Ca	1348 ± 90	495 ± 94	486.9 ± 86.1	1583 ± 44	7194 ± 20	8296 ± 160
Cu	2.5 ± 0.3	2.58 ± 0.025	2.9 ± 0	1.15 ± 0.05	0.4 ± 0	1.6 ± 0
Fe	1.7 ± 0.005	22 ± 3.2	10.06 ± 2.54	4.2 ± 0.1	2.56 ± 0.31	169.5 ± 3.5
Zn	0.36 ± 0.045	0.64 ± 0.01	0.43 ± 0.09	0.2 ± 0.04	0.07 ± 0.02	0.31 ± 0.1
Mn	2 ± 0.3	7.34 ± 0.15	7.85 ± 2.05	1.75 ± 0.15	0.8 ± 0	15.55 ± 0.05
B	2.9 ± 0.7	1.8 ± 0.15	1.765 ± 0.005	1.84 ± 0.06	3.79 ± 0.16	98.3 ± 2.7
S	3.9 ± 1.5	13.3 ± 5.4	3.8 ± 1.9	2.7 ± 0.2	11.4 ± 0.6	488.5 ± 12.5
Na	357 ± 123	89.6 ± 20.7	8.04 ± 1.15	16.1 ± 0	42.51 ± 3.45	982 ± 40

^*a*^ EC, electric conductivity; OM, organic matter.

### Metagenomic shotgun sequencing

DNA extraction was carried out as previously described (38, 40). Briefly, total DNA from soils was extracted from 10 g of soil using the NucleoSpin Food kit (Macherey-Nagel), following the manufacturer’s instructions. DNA obtained from soil samples was pooled to obtain one representative DNA sample per site. The libraries were prepared using the Nextera DNA Flex library preparation kit (Illumina) following the manufacturer’s user guide. The initial concentration of DNA was evaluated using the Qubit dsDNA HS Assay Kit (Life Technologies). 50 ng DNA was used to prepare the libraries. Following the library preparation, the final concentration of the libraries was measured using the Qubit dsDNA HS Assay Kit (Life Technologies), and the average library size was determined using the Agilent 2100 Bioanalyzer (Agilent Technologies). Sequencing was carried out by MR DNA (www.mrdnalab.com, Shallowater, TX, USA) on a Miseq platform (Illumina, San Diego, CA) with an overlapping 2 × 150 bp configuration, obtaining an average 20 Gb of data per metagenome. Table S2 provides a summary of sequencing information.

### Assembly, binning, and dereplication of MAGs

Metagenomes were assembled with IDBA-UD 1.1.1 using pre-correction step-activated, k-mers from 64 to 124 with 20 k-mers jumps (six processing steps) after quality checking and adaptor trimming on raw reads (41). Annotation of the six metagenomes was carried out using Prodigal v2.6.3 (42). All samples were assembled separately and scaffolds were binned using CONCOCT according to GC-content, k-mers frequency (tetramers), and abundance of reads across samples (43). Hybrid assemblies were performed following the tool’s protocol with modifications according to Alneberg et al. (44). Information on metagenomic assemblies is available in Table S3. Bin completeness and contamination were evaluated by checking for single-copy gene sets with CheckM 1.0.13 (45). Accepted bins were re-assembled into MAGs using Velvet 1.2.10 (46). Dereplication of redundant MAGs was performed using dRep v2.2.4 with default parameters (47). MAGs were considered redundant if they shared more than 95% whole-genome ANI. Taxonomic assignment of MAGs was executed against the Genome Taxonomy Database with the GTDB tool kit 1.6.0 (48, 49). The raw abundance of MAGs was determined as the mapping coverage of reads obtained from the six metagenomes sequenced. Mapping was carried out using Bowtie2 2.3.4.3 and coverage was calculated with BEDTools 2.17.0 (50, 51). Relative abundances correspond to individual raw abundances over the sum of MAGs kept after quality filters (minimum completeness: 70%, maximum contamination: 10%) per site. To consider a genome as “present” in each sampled site, a cut-off for relative abundance of 1% was set. Table S4 provides a summary of genomic information of MAGs.

### Comparative analyses on functional information of BGCs

Non-redundant, quality-filtered MAGs (*n* = 43) were considered for functional analyses. Prediction and annotation of BGCs, and their comparison against the Minimum Information about a Biosynthetic Gene Cluster (MIBiG) repository were performed with antiSMASH 5.1.2 webserver in relaxed mode and KnownClusterBlast option on (52, 53). The tool’s basic options also retrieve gene classifications according to specialized metabolite categories of ortholog groups (smCOGs) constructed upon hidden Markov models of protein families (PFAM) (54). The “overview” output tables were fused and BGCs of lengths shorter than 5,000 bp were filtered out (Table S5), while the genbank output files were converted into GFF format with the script bp_genbank2gff3.pl of Bio::DB::GFF module calling the flags --split and --noinfer, and then parsed (Table S6) (55). For downstream analyses, these two databases were transformed into presence/absence matrices of (i) types of core biosynthetic capabilities of BGCs per genome and (ii) regulatory, transport, and transposase smCOGs in BGCs per biosynthetic classes as defined in the work of Navarro-Muñoz et al. (56) (Tables S8 and S9). Distances of the scaled matrices were calculated with the Manhattan metric; then, hierarchical clustering was conducted in R with the base function “hclust,” linked by Ward’s minimum variance method, plotted with “pheatmap”, and styled with Inkscape (57, 58). Specialized metabolite redundancy was explored throughout BiG-SCAPE 1.1.1 which groups BGCs into gene cluster families (GCFs) with highly similar predicted metabolite chemotypes (56). The flags --mix, --mibig, and --hybrids-off were called. Default “auto” mode accounts for comparisons between complete and fragmented BGCs by choosing the “glocal” instead of “global” option when at least one of them has one or both of its neighborhoods located at a contig edge. Sequence similarity networks were visualized with Cytoscape 3.10.1 (59). Manual inspection of GCFs with at least two members was conducted throughout the analysis of PFAM domains of reference BGCs; for conversion into GO terms, we used the “pfam2go” function of the ragp package in R (Table S9) (60, 61).

## RESULTS

### Environmental heterogeneity across the Talabre-Lejía altitudinal gradient

Environmental conditions along the TLT (Fig. 1) have been described previously (32, 33, 37). Briefly, site S1 corresponded to a pre-puna ecosystem with the lowest levels of precipitations and the highest mean annual temperature (Table S1). Soil samples from S1 were rich in K and Na while low measurements for NH_4_ and NO_3_ were obtained (Table 1). Site S2 corresponded to a puna ecosystem, where temperature and precipitation conditions were less stressful in comparison to the pre-puna and steppe belts. S2 was characterized by a high content of organic matter (OM), copper, and zinc (Table 1). The remaining sites were located above 4,300 m.a.s.l., in an area nested at the summit of the Lascar Volcano, the most active volcano of the northern Chilean Andes (Fig. 1). Sites S3, S4, S5, and S6 were located at the same altitude, corresponding to steppe ecosystems, and were exposed to higher precipitations and lower temperatures than sites S1 and S2. Sites S3, S4, and S5 were selected based on differences in soil pH values, driven by calcium and boron contents (Table 1). Soil samples from site S6 were collected from the shore of the Lejía Lake (Fig. 1) and, consistent with descriptions provided by Demergasso et al. (62), exhibited an elevated electric conductivity (1.94 mS/cm), and up to 181-, 122-, 66-, and 56-folds of sulfur, sodium, iron, and boron, respectively, in comparison with soils from sites S3, S4, and S5. Finally, the highest measurement for NO_3_ was measured in S6 (20.5 ± 5.5 mg/kg, Table 1).

### Overview of the genomic data set

MAGs were generated from the shotgun sequencing data of soil samples from sites S1 to S6. A total of 832 × 10^6^ good-quality reads were obtained after filtering 905 × 10^6^ metagenomic reads (Table S2). All samples were assembled separately and as pairs of samples when they were spatially close, such as sites S3 +S4 and S4 +S5. This protocol increases the probability of assembling suitable scaffolds using sequences shared between samples. Total assembly lengths of metagenomes ranged from 0.45 to 1.64 GB. A summary of statistics (i.e., total base pairs, number of scaffolds, average length, and N50) for each metagenome assembly is available in Table S3. The 43 MAGs that passed the quality thresholds for completeness (>70%) and contamination (<10%) were considered for functional analyses. Based on completeness and contamination indices, MAGs were classified as high (*n* = 13), good (*n* = 17), or medium (*n* = 17) quality draft genomes (Table S4). On average, quality-filtered MAGs yielded 3.38 × 10^3^ bp in length and a N50 of 32.6 × 10^3^ bp. The taxonomy of MAGs, assigned with GTDB-tk, revealed that *Acidobacteriota, Proteobacteria*, and *Actinobacteriota* were dominant, together recruiting 72% and 80% of overall richness and relative abundance, respectively (Table S4). MAGs from these three phyla were detected at sites S1, S2, S3, S4, and S5, whereas all MAGs from site S6 [*Proteobacteria* (*n* = 7), *Bacteroidota* (*n* = 4), *Desulfobacterota* (*n* = 3), *Spirochaetota* (*n* = 1)*,* and *Verrucomicrobiota* (*n* = 1)] were found to be exclusive to that sample when spatial distribution of abundant MAGs was examined (see Methods). Our data showed that most MAGs were specific to a single site (*n* = 37) and that in all sites there is at least one site-specific MAG (Table S4).

### Biosynthetic potential of MAGs

**Fig 2:**
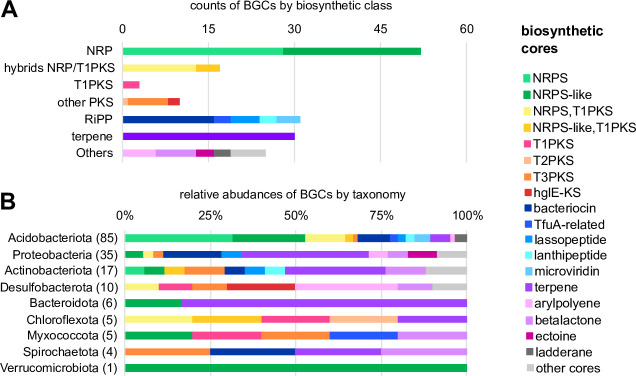
Biosynthetic potential of MAGs. (**A**) Counts and (**B**) relative abundance of BGCs longer than 5,000 bp (*n* = 168) according to the biosynthetic classes of their core enzymes as defined by Navarro-Muñoz et al. (56) and to the phyla of MAGs in which they were predicted, respectively.

We detected at least one BGC in 38 of the 43 recovered MAGs. The data set comprised 168 BGCs (Fig. 2) out of which 147 were identified as single candidate clusters (Table S5), meaning that their core is constituted by genes encoding for a unique type of specialized enzyme. More than half of BGCs (*n* = 85) were found in MAGs belonging to the *Acidobacteriota* phylum, where four of these MAGs accounted for almost a third of the identified BGCs: MAG009 (*n* = 23), MAG052 (*n* = 16), MAG008 (*n* = 11), and MAG007 (*n* = 8), with genome sizes ranging from 3.98 Mb to 6.27 Mb (Table S4). In particular, MAG008 belonging to the *Pyrinomonadaceae* family harbored the maximum number of different types of biosynthetic cores (*n* = 9), including five different types of RiPPs, and contained the longest BGC of the data set (131 kb), with a 50% of gene similarity to the reference BGC for nostopeptolide A2 (BGC0001028, *nosA*, *nosB*, *nosC,* and *nosD*, Fig. S3). The functional diversity of BGCs includes 22 types of core genes detected with fifth version of antiSMASH. Predominant biosynthetic classes of BGCs were non-ribosomal peptides (NRP), post-translational modified peptides (RiPP), and terpenes (Fig. 2), mainly predicted in MAGs of the *Acidobacteriota* and *Proteobacteria* phyla (Fig. S2). Regarding functional novelty, our analysis showed that only 11.3% of BGCs (*n* = 19) had a match of ≥60% when homolog genes were searched against the established set of known BGCs available in MIBiG 2.0 (columns “knownClusterBlast match” and “gene similarity” in Table S5). However, only one of these BGCs, found in MAG005, was linked to a verified compound according to BiG-SCAPE’s similarity indices, the ectoine under the accession “BGC00008600” (see column “MIBiG match” in Table S5). With respect to gene composition, the 168 BGCs contained a total of 2,577 coding sequences, of which 978 have functional annotations. Among them, 303 coding sequences were detected as “core” for biosynthesis and 675 as accessory genes (column “gene type” in Table S6). Following the smCOG nomenclature, this last group included 446 genes annotated as “additional biosynthetic” and 229 were assigned to the “transport,” “regulatory,” or “other” functional categories (column “gene kind” in Table S6).

### Functional redundancy of specialized metabolites predicted in MAGs

**Fig 3:**
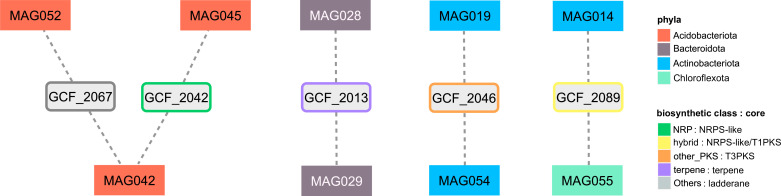
Functional redundancy of specialized metabolites. Sequence similarity networks of GCFs with at least two members as delivered by BiG-SCAPE. Rectangular color-filled nodes are MAGs, rounded gray nodes with colored borders are GCFs, and edges are the shared BGCs.

A sequence similarity network analysis revealed that 158 of the 168 BGCs were singletons, meaning that the vast majority of BGCs retrieved (94.1%) encode for structurally unique metabolites and constitute the only members of their respective gene cluster family (GCF, see Materials and Methods). The other 10 BGCs were grouped into five GCFs with two or more members, constituting a functionally redundant subset (column “GCF ID” in Table S5). Taking advantage of the binning step, we fused GCFs by genomic origin and generated four final networks linking nine MAGs that were predicted to encode highly similar specialized metabolites (Fig. 3). We observed that three of these networks linked MAGs of the same taxonomic families according to the GTDB. Namely, MAGs from *Acidobacteriota* linked by GCF_2042 and GCF_2067 belong to *Pyrinomonadaceae*, MAGs from *Bacteroidota* to *Cryomophaceae* (GCF_2013), and MAGs from *Actinobacteriota* to *Rubrobacteraceae* (GCF_2046). Only GCF_2089 was found in MAGs from two different phyla (*Actinobacteriota* and *Chloroflexota*). Manual inspection of PFAM domains from the reference BGC from each GCF highlighted GCF_2067 and GCF_2089 as possible antimicrobial compounds (Table S9).

### Occurrence patterns in specialized genomic repertoires

**Fig 4:**
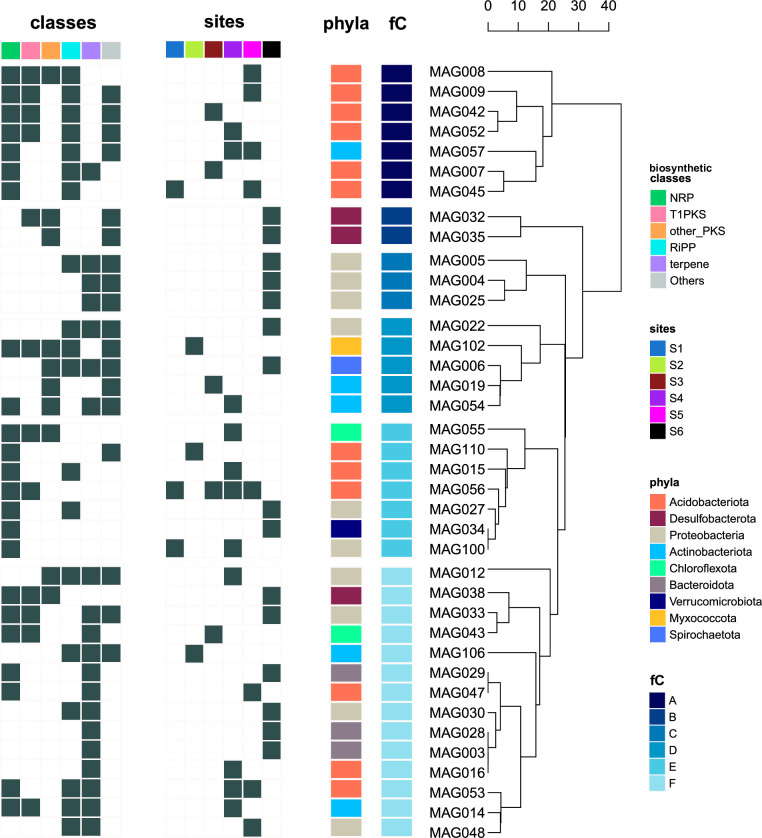
Occurrence patterns in specialized genomic repertoires. Hierarchical clustering of MAGs with predicted BGCs (*n* = 38) based on the presence or absence of types of core biosynthetic enzymes (*n* = 22) over 125 observations. Sites are colored if relative abundance was above 1%; from left to right: S1 to S6. Single boxes show taxonomical phyla according to GTDB and the names of functional clusters (fC).

Hierarchal clustering of MAGs with regards to the types of core genes they encode (Table S7) allowed us to divide the data into six functional groups (Fig. 4). The seven MAGs from Cluster A were characterized by encoding, on average, for six different types of core enzymes including bacteriocins (from the RiPP class) along with the type I polyketides (T1PKS) and NRP classes, while the rest of the data set averaged three different types of biosynthetic cores. Taxonomical assignments revealed that five members of this cluster belonged to the *Pyrinomonadaceae* family from the *Acidobacteriota* phylum. Cluster B consists of two genomes belonging to *Desulfobacterota,* in which heterocyst glycolipid synthase-like PKS (hglE-KS) were detected, representing the functional signature that distinguished MAG032 and MAG035 from other genomes. The presence of ectoines appears to be enough to classify three MAGs in Cluster C. Taxonomical assignments indicated that MAG004 and MAG025 belonged to the *Gammaproteobacteria* class, while MAG005, which also harbors a bacteriocin-encoding BGC, belonged to *Alphaproteobacteria*. Regarding spatial distribution, the two MAGs in Cluster B (*Desulfobacterota*) and the three in Cluster C (*Proteobacteria*) exclusively inhabited site S6 (Fig. 4). The other three functional groups (Clusters D, E, and F) showed no obvious relationship between composition of specialized biosynthetic potential and taxonomy or geographic distribution. Beta-lactones and T3PKS characterize Cluster D while enzymes classified as NRPS-like characterize Cluster E; however, none of these specialized features constitute a functional signature as they are not unique to a comprehensive subset of MAGs. Cluster F comprises more than a third of the genomic data set (*n* = 14) from miscellaneous phyla, of which all but one harbor BCGs that encode for terpenes (Table S7).

### Potential functional links among biosynthetic classes and accessory genes of BGCs

**Fig 5:**
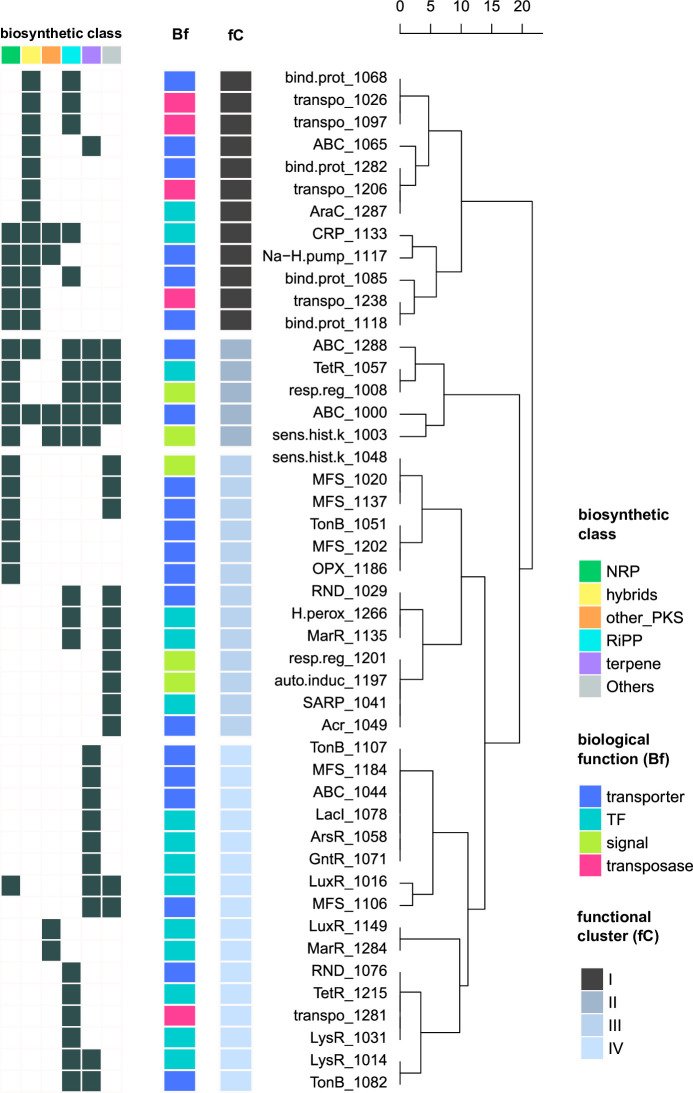
Potential functional links among biosynthetic classes and accessory genes of BGCs. Hierarchical clustering of transport, regulatory, and transposase smCOGs (*n* = 46) assigned to accessory genes based on their presence or absence in the different biosynthetic classes (*n* = 6)) over 88 observations. Leaves are labeled by short names and ID numbers of smCOGs. TF: transcription factor, ABC: ATP-binding cassette transporter, auto.induc: unspecified autoinducer, CRP: catabolite repressor protein, bind.prot: binding protein-dependant transport systems, MFS: major facilitator superfamily, Na-H.pump: sodium-hydrogen exchanger, sens.hist.k: sensor histidine kinase, resp.reg: unspecified response regulator, RND: resistance, nodulation, and cell division superfamily of transporters, OPX: outer membrane polysaccharide export, H.perox: hydrogen-peroxide sensitive repressor, Acr: multidrug efflux pump AcrB/AcrD/AcrF, SARP: Streptomyces antibiotic regulatory protein, transpo: transposase.

To evaluate the functional relationship between classes of BGCs (n=6) and accessory genes detected in the data set, we analyzed the annotated functions of these tailoring genes located at the neighborhoods of the biosynthetic cores, using the smCOG classification (see Methods, Table S6). We selected 46 categories of annotations involved in four types of biological functions (“membrane transport” from “transport”, “transcription factor” and “signaling” from “regulatory”, and “transposase” from “other” smCOGs, Table S8) and then carried out a hierarchical clustering. The results revealed that four out of five transposase-encoding genes and all binding protein-dependent transporter domains were associated with NRP/T1PKS hybrid BCGs and shaped the signature of Cluster I (Fig. 5). The fifth transposase with a different annotation, the insertion sequence IS407 (Orf A), was only detected among accessory genes of RiPP BCGs (Cluster IV). Regulatory genes such as CRP and AraC transcriptional regulators were also identified in Cluster I. In Cluster II, genes annotated as ATP-binding cassette transporters (“ABC_1288” and “ABC_1000”), tetracycline transcriptional regulators (“TetR_1057”), sensor histidine kinases (“sens.hist.k_1003”), and response regulators (“resp.reg_1008”) were associated with different biosynthetic classes, including NRP, RiPP, and terpenes. However other accessory genes with related functional annotations (“ABC_1044,” “ABC_1065,” ”sens.hist.k_1048,” “resp.reg_1201,” and “TetR_1215”) were associated with a unique biosynthetic class. Cluster III was dominated by BGCs predicted to encode for various specialized metabolites wrapped in the “Others” class while lacking any type of polyketides and terpenes. In addition, accessory genes related to antibiotics and oxidative stress resistance were also found to be associated with this conglomerate of rarer types of BGCs and the RiPP class including annotations for the resistance, nodulation, and cell division superfamily of transporters (RND), the multidrug efflux pump AcrB/AcrD/AcrF (Acr), the *Streptomyces* antibiotic regulatory protein (SARP), the MarR family of transcriptional regulators, and a hydrogen peroxide repressor. Lastly, most smCOGs in Cluster IV were associated with the RiPP and/or terpene classes (Fig. 5). LysR was found exclusively in RiPP-encoding BGCs. Notably, genes encoding the transcriptional regulators LacI, GntR, and ArsR and two of the three TonB-dependent siderophore receptors were only detected among accessory genes of terpene BGCs (Table S8).

## DISCUSSION

Deserts comprise a wide range of ecological niches where microbial communities have evolved to produce multiple bioactive compounds in the struggle to survive under oligotrophic conditions (34, 63). Many studies employing chemical profiling and culturing techniques toward the prospection of new drugs have identified the Atacama Desert as a promising source of novel compounds. In fact, by exploring isolated bacteria from the Atacama Desert, dozens of specialized metabolites exhibiting antimicrobial (e.g., tatiomycin and asenjonamides), antitumoral (e.g., atacamycin), or antiviral (e.g., lentzeosides) properties have been described (30, 64–67). To improve the bioprospecting of natural products in this extreme environment, the present study implemented a metagenomic approach to explore the taxonomic and functional composition of BGCs from uncultivated extreme microbes (34, 68). Our results assessing spatial distribution showed that MAGs with a relative abundance greater than 1% are mostly exclusive to a single site, suggesting that their metabolic features might be the result of site-specific adaptations driven by a tight combination of environmental stressors. In particular, the 16 MAGs from the Lejía Lake’s shore were only detected at that location, which is susceptible to explanation by the unique physicochemical and nutritional conditions observed in S6 (40, 62, 69).

In agreement with predominant biosynthetic classes detected in other large-scale explorations performed in natural settings, the most common types of BGCs identified in this study were classified as NRP, RiPP, and terpene (12, 70–72). While a few BGCs matched more than 60% of genes from any other BGC of known function, only one was predicted to biosynthesize a metabolite that would actually have a similar chemical structure to the reference from the MIBiG repository, underscoring the limited knowledge on specialized metabolites in soil microbiomes. Furthermore, the majority of the BGCs in our data set constituted singletons, suggesting that the still-largely uncharacterized biosynthetic potential of the Atacama Desert is divergent even for microorganisms that have evolved under the same extreme environmental conditions (73, 74). On the other hand, 23.7% of MAGs encoded at least one BGC that was also predicted in another MAG. These results imply that, even though most BGCs of the TLT belonged to species-specific repertoires, there is a functionally redundant fraction, shared between organisms inhabiting this Andean ecosystem. In general, MAGs that share BGCs are members of the same taxonomic family, supporting recent findings that argue in favor of understanding BGCs as phylogenetically conserved functional traits (75–77). Furthermore, studies employing comparative approaches have reported BGCs to be circumscribed to the taxonomic ranks of phylum and order in communities from soil biocrusts and Antarctic soils, respectively, and to populations in dozens of isolates from *Aspergillus flavus* and ant-associated *Pseudonocardia* (78–81).

Through an ordination analysis of MAGs, we observed occurrence patterns in BGCs according to the types of core enzymes they encode for. We found that some metabolic features were associated with taxonomies. For example, the enriched biosynthetic capabilities of *Acidobacteriota* in Cluster A, characterized by NRP/T1PKS hybrids and bacteriocins, are consistent with reports pointing to that taxon as one of highly specialized biosynthetic diversity (70, 80, 82, 83). Sometimes, genomic specialized repertoires were also indicative of the geographic origins of MAGs. Specifically, the functional signatures of heterocyst glycolipids (hglE-KS) in Cluster B and of ectoines in Cluster C are strictly associated with soil bacteria from the Lejía Lake. In particular, the two *Desulfobacterota* in Cluster B are likely to be nitrogen fixators through hglE-KS mechanisms (84). This is consistent with a study highlighting the role of this phylum in nitrogen cycling and another report on 52 *Desulfobacterota* MAGs encoding for hglE-KS-encoding BGCs in sulfidic and mangrove sediments of coastal ecosystems, respectively (85, 86). We speculate that the high contents of NO_3_ measured at S6 are likely to drive the specialized metabolism of these microorganisms toward alternative mechanisms for the acquisition of organic nitrogen, typically described for photosynthetic cyanobacteria (84). This observation enables us to further surmise that some members from *Desulfobacterota* might constitute keystone taxa by providing nitrogen-containing compounds to the microbial community through cross-feeding (87). On the other hand, the three members of *Proteobacteria* in Cluster C are likely to maintain turgor and avoid cytoplasmic dehydration by applying a “salt-in” strategy through the biosynthesis of ectoines, a well-known adaptation to cope with drought and hyperosmotic conditions (88). The extreme saline conditions of the Lejía Lake can be evidenced by the high electric conductivity and Na contents measured in S6. These findings suggest that encoding for these types of machineries might be understood as biogeographic signals, tightly adapted to survive at the shore of a brine lagoon, a hostile environment subjected to permanent oxidative, osmotic, and nutritional stress ensured by high UV irradiance, high salinity, and high contents of sulfur and boron, among other factors (62).

Lastly, the analyses on functional annotations of accessory genes showed some degree of association with the biosynthetic classes of BGCs. For instance, transposase domains in Cluster I suggest that NRP/T1PKS hybrids and some RiPPs like lanthipeptides and microviridins could be susceptible to undergo DNA rearrangements involving gene fusions, duplications, and domain shuffling events and, thus, functional diversification (17). Also, our results linking all smCOGs annotated as binding protein-dependent transporters with NRP/T1PKS hybrids imply that this class of BGCs could interact with extracellular ligand/solute complexes. Regarding regulatory functions of accessory genes, while regulators classified in the LuxR and TetR families showed some functional promiscuity by being annotated in BGCs from four different biosynthetic classes, other major regulators involved in numerous cellular processes like the Lacl, GntR, and TetR families were mainly restricted to RiPPs or terpenes. Notwithstanding, we acknowledge that to gain better insights into the many different possible activities of transporters, regulators, and other specialized enzymes in desert environments, considerations about using complementary databases, larger data sets, and long read-sequencing technologies are needed for future efforts (80, 81).

## Data Availability

Nucleotide sequences of MAGs are deposited at the NCBI database under the BioProject accession PRJNA291433. Files containing sequences and annotations of BGCs can be downloaded at https://github.com/cmandreani/BGCdataset_AtacamaTLT/ (DOI: 10.5281/zenodo.10680981).

## SUPPLEMENTAL MATERIAL

Tables S1-S3 and Figures S1 and S2 msphere.00192-24-s0001.docx Sampled data and BGC analysis.

Tables S4 to S9 msphere.00192-24-s0002.xlsx Genomics and BGC data.

ASM does not own the copyrights to Supplemental Material that may be linked to, or accessed through, an article. The authors have granted ASM a non-exclusive, world-wide license to publish the Supplemental Material files. Please contact the corresponding author directly for reuse.
