# Supplementary material for: Biosynthetic gene clusters from uncultivated soil bacteria of the Atacama Desert
Source: mSphere. 2024 Sep 17;9(10):e00192-24. doi: 10.1128/msphere.00192-24 (PMC11520301; doi:10.1128/msphere.00192-24)
Supplement: Tables S1-S3 and Figures S1 and S2 — Sampled data and BGC analysis. [file msphere.00192-24-s0001.docx]

# **Biosynthetic gene clusters from uncultivated soil bacteria of the Atacama Desert**

Constanza M. Andreani-Gerard^a,b,c^, Verónica Cambiazo^a,b^, Mauricio González^a,b^#

^a^ Millennium Institute Center for Genome Regulation (CRG).

^b^ Bioinformatic and Gene Expression Laboratory, INTA – Universidad de Chile.

^c^ Center for Mathematical Modelling – Universidad de Chile.

#Please address correspondence to Mauricio González, [mgonzale@inta.uchile.cl](mailto:mgonzale@inta.uchile.cl).

**SUPPLEMENTARY INFORMATION**

Geographical information:

Supplementary Tables 1 page 2

Metagenomic information:

Supplementary Tables 2 and 3 page 3

Functional information:

Supplementary Figure 1 page 4

Supplementary Figure 2 page 5

Additional information

Supplementary Tables 4 – 9 excel file

**GEOGRAPHICAL INFORMATION:**

**Supplementary Table 1**. Geographical information of the Talabre-Lejía transect. Data was extracted from Díaz *et al.* (2016). MAT: mean annual temperature, MAP: mean annual precipitations, m.a.s.l.: meters above sea level, δ15N: foliar N isotopic values, ALV: alluvial, IGN: ignimbrite, SAN: sandy alluvial, VOL; volcanic, QFL: late quaternary fluvial terrace.

| Geographical data | S1 | S2 | S3 | S4 | S5 | S6 |
| --- | --- | --- | --- | --- | --- | --- |
| Altitude (m.a.s.l.) | 2870 | 3870 | 4480 | 4480 | 4480 | 4314 |
| ID of sites | TLT18 | TLT08 | TLT01 | TLT01 | TLT01 | Lejía Lagoon |
| Latitude S | -23.28868° | -23.32856° | -23.50205° | -23.50333° | -23.50338° | -23.50433° |
| Longitude W | -67.94587° | -67.79890° | -67.72377° | -67.70666° | -67.70636° | -67.69744° |
| Vegetation belt | Pre-puna | Puna | Steppe | Steppe | Steppe | Steppe |
| MAT (°C) | 11.8 | 6.9 | 4.2 | 4.2 | 4.2 | 8.5 |
| MAP (mm/year) | 15.0 | 75.1 | 161.9 | 161.9 | 161.9 | 161.9 |
| Soil regolith | QFL | SAN, VOL | ALV, IGN | ALV, IGN | ALV, IGN | ALV, IGN |
| Mean δ15N | 6.0 | 5.4 | 2.1 | 2.1 | 2.1 | 2.1 |
| Aridity (De Martonne index) | 0.7 | 4.4 | 11.4 | 11.4 | 11.4 | 11.4 |

**METAGENOMIC INFORMATION:**

**Supplementary Table 2**. Metagenomic sequencing information.

|  | Raw | | | Filtered | | |
| --- | --- | --- | --- | --- | --- | --- |
| Sample | Total Reads | Data (Gb) | Avg. read length (bp) | Total Reads | Data (Gb) | Avg. read length (bp) |
| S1 | 183,039,130 | 26.7 | 145.8 | 162,035,390 | 23.2 | 143.4 |
| S2 | 183,616,404 | 26.8 | 146.2 | 166,329,234 | 24.0 | 144.2 |
| S3 | 160,145,256 | 23.5 | 146.5 | 153,290,713 | 21.0 | 137.0 |
| S4 | 147,991,252 | 22.0 | 148.8 | 141,107,489 | 19.5 | 138.2 |
| S5 | 134,596,770 | 20.0 | 148.8 | 127,793,777 | 17.7 | 138.3 |
| S6 | 96,496,520 | 14.4 | 149.2 | 81,854,686 | 11.9 | 145.7 |
| Total | 905,885,332 | 133.4 |  | 832,411,289 | 117.3 |  |

**Supplementary Table 3**. Metagenomic assembly information.

| Sample | Total length (Mb) | Number of scaffolds | Avg. length (pb) | Max. length  (pb) | N50  (pb) | L50 | used reads  (%) | GC  (%) |
| --- | --- | --- | --- | --- | --- | --- | --- | --- |
| S1 | 460 | 578,420 | 795 | 231,815 | 851 (75%) | 436,427 | 17.8% | 67.0% |
| S2 | 655 | 727,989 | 900 | 174,227 | 988 (80%) | 579,801 | 21.0% | 65.4% |
| S3 | 823 | 893,733 | 920 | 306,936 | 1,283 (87%) | 776,204 | 38.3% | 67.3% |
| S4 | 830 | 892,653 | 929 | 459,495 | 1,334 (88%) | 788,942 | 33.2% | 66.6% |
| S5 | 678 | 714,882 | 948 | 1,145,089 | 1,384 (90%) | 640,269 | 32.2% | 64.7% |
| S3 + S4 | 1,542 | 1,656,500 | 931 | 1,145,083 | 1,343 (89%) | NA | 34.5% | 65.7% |
| S4 + S5 | 1,685 | 1,832,894 | 919 | 567,355 | 1,290 (87%) | NA | 36.6% | 66.9% |
| S6 | 592 | 640,971 | 924 | 368,304 | 1,426 (89%) | 573,308 | 48.3% | 53.8% |

**FUNCTIONAL INFORMATION:**


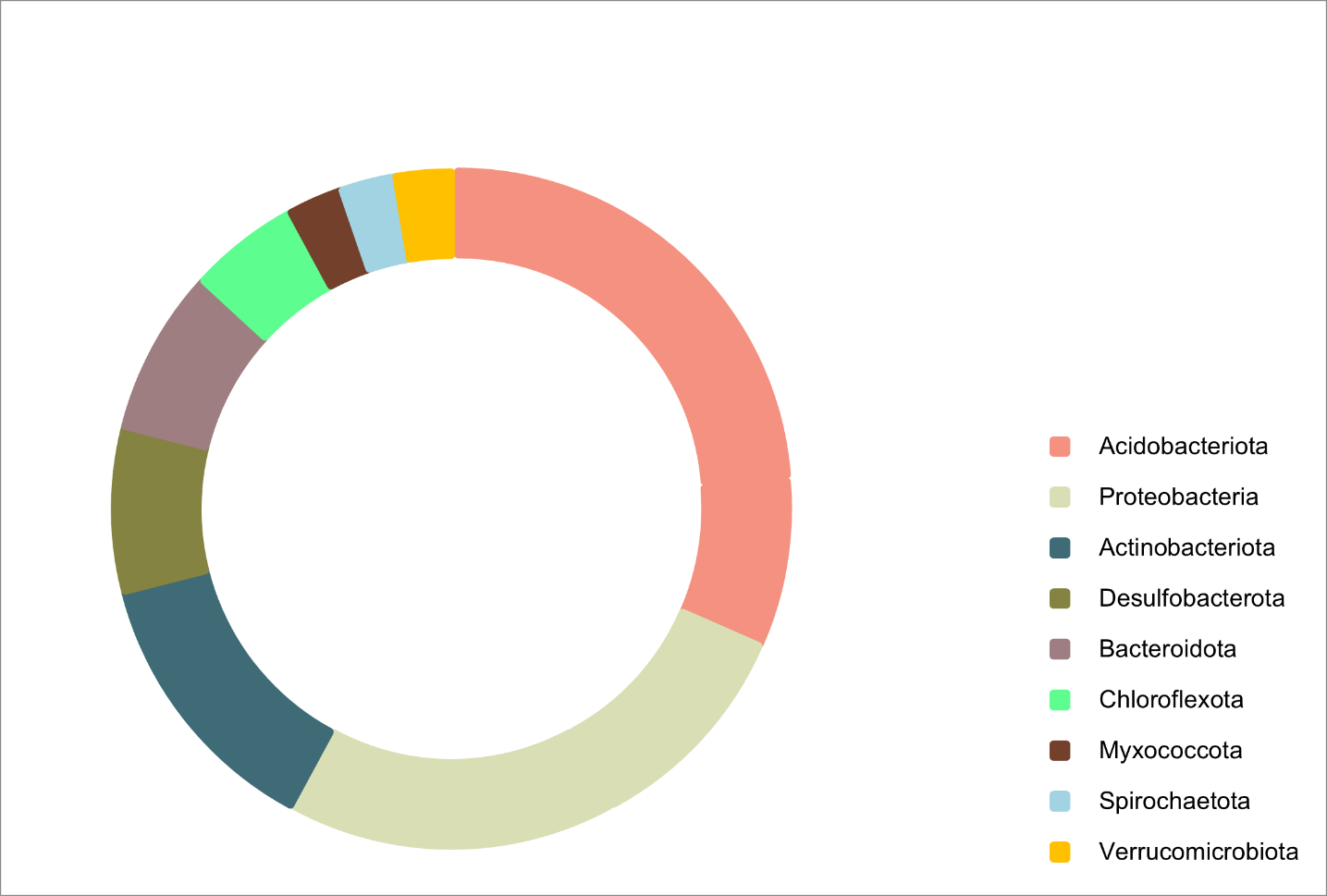

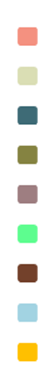
**Supplementary Figure 1.** (A) Dataset of MAGs with predicted BGCs (n=38) colored by taxonomy. The outer and inner circles correspond to assignments of phyla and classes, respectively. (B) Percentual contributions of taxonomical classes to the BGC collection (n=168) grouped by biosynthetic class of predicted specialized metabolites.

**Phylum Class**

**A**

**B**

**Supplementary Figure 2**. (A) antiSMASH output for region 2 detected in MAG008, a complete hybrid region of 131,091 bp matching all cores of (B) the reference BGC isolated from Nostoc *sp*. GSV224 responsible for the production nostopeptolide A2. Black filled arrows: genes of the query with homologs in MiBiG, red: core genes, blue: transport-related genes, green: regulatory genes, pink: genes ‘additional’ for biosynthesis, grey: genes annotated with ‘other’ smCOGs.
(C) Structure and (D) properties of the predicted compound of (B) according to The Natural Products Atlas (van Santen JA, Jacob G, *et al.* (2019). *ACS Cent. Sci.* 5: 11, 1824–1833).

10 kb


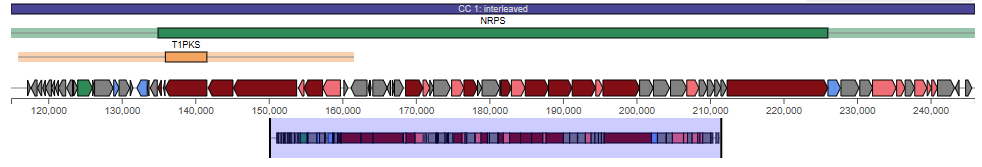

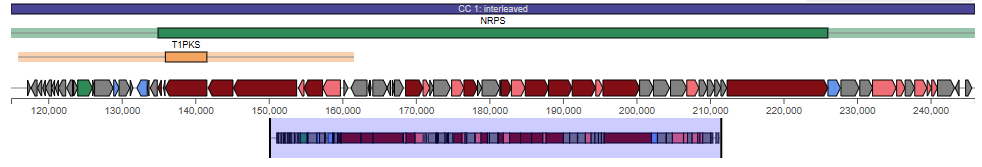


**A**


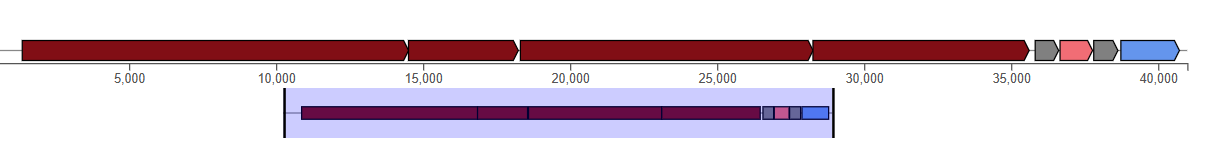

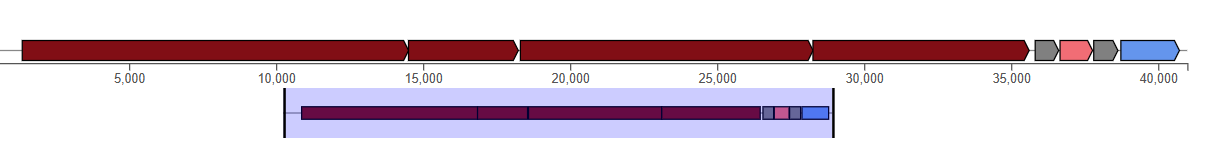


nosA

nosB

nosC

nosD

**B**


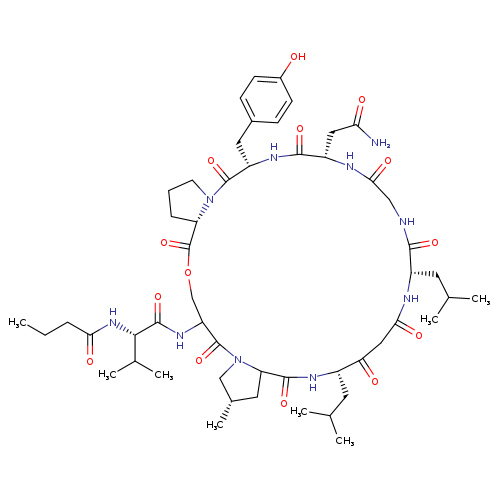


**C**


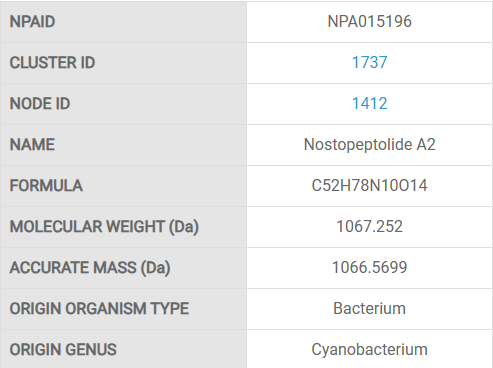


**D**
